# Supplementary material for: Salivary DNA Loads for Human Herpesviruses 6 and 7 Are Correlated With Disease Phenotype in Myalgic Encephalomyelitis/Chronic Fatigue Syndrome
Source: Front Med (Lausanne). 2021 Aug 6;8:656692. doi: 10.3389/fmed.2021.656692 (PMC8378328; doi:10.3389/fmed.2021.656692)
Supplement: Supplementary file 1 [file Data_Sheet_1.pdf]

Supplementary Tables

Supplementary Table 1. Primer and probe sequences. Assays were performed as duplex assays by incorporation of FAM or HEX reporter labelled probes: HSV1& HSV2; EBV & CMV; HHV6A & HHV6B; HHV7 & HHV8. VZV was performed as a single assay.

Target	Forward Primer	Reverse Primer	Probe
HSV-1 (gD_U56)	CGGCCGTGTGACACTATCG	CTCGTAAAATGGCCCCTCC	FAM-CCATACCGACCACACCGACGAACC-BHQ1
HSV-2 (gG_U54)	CGCTCTCGTAAATGCTTCCCT	TCTACCCACAACAGACCCACG	HEX-CGCGGAGACATTCGAGTACCAGATCG-BHQ1
VZV (DNA polymerase)	CGGCATGGCCCGTCTAT	TCGCGTGCTGCGGC	FAM-ATTCAGCAATGGAAACACACGACGCC-BHQ1
EBV (BALF5)	CGGAAGCCCTCTGGACTTC	CCCTGTTTATCCGATGGAATG	FAM-TGTACACGCACGAGAAATGCGCC-BHQ1
CMV (UL55)	TGGGCGAGGACAACGAA	TGAGGCTGGGAAGCTGACAT	HEX-TGGGCAACCACCGCACTGAGG-BHQ1
HHV-6A (U67/68)	TTCCGGTATATGACCTTCGTAAGC	GATGTCTCACCTCCAAATCTTTAGAAAT	6FAM-CATTATATATCGAATCTGACGCTACCTTCCG-BHQ1
HHV-6B (U67/68)			HEX-ACATTATATGTCGAACTTGACACTACCTTCCG-BHQ1
HHV-7 (gB_U39)	TTTCCTGTGACAAAAGAAGCAGTTA	ATCCCACACGCTTTACGGG	FAM-TTCCTGCGCAATAAAGTGAAAACTGTTAGCATT-BHQ1
HHV-8 (MCP_Orf25)	CCACCCTCGAATGCACAAC	GTCGGGATCGGGAAAAGCT	HEX-CCACCCAGTCAGCCCAGGCACTAAAC-BHQ1

Supplementary Table 2. Participants Characteristics of HHV DNA positivity from Plasma and PBMC

	Participant number (n=)		Gender (n=)	Age, median (IQR)	From DNA positive sample Detected HHV	Age range (n=)
**HC**	Total	76	F (62)/ M (14)	44.0 (32.3-52.8)		
	DNA negative	63	F (49)/ M (14)	46.0 (35.0-54.0)		
	DNA positive	13	F (13)	32.0 (24.0-43.0)		
					EBV	18-29 (1)
						41-60 (2)
					HHV-6A	30-40 (1)
					HHV-6B	18-29 (1)
						30-40 (1)
					HHV-7	18-29 (3)
						30-40 (1)
						41-60 (2)
					CMV+HHV-7	30-40 (1)
**ME/CFS _MM**	Total	99	F (78)/ M (21)	43.0 (35.0-53.0)		
	DNA negative	88	F (68)/ M (20)	43.5 (37.0-52.0)		
	DNA positive	11	F (10)/ M (1)	32.0 (25.0-53.0)		
					EBV	41-60 (2)
					HHV-7	18-29 (4)
						30-40 (2)
						41-60 (3)
**MC/CFS _SA**	Total	33	F (25) / M (8)	48.0 (34.0-53.5)		
	DNA negative	23	F (15) / M (8)	49.0 (39.0-53.0)		
	DNA positive	10	F (10)	36.5 (30.5-55.3)		
					EBV	41-60 (1)
					HHV-7	18-29 (1)
						30-40 (4)
						41-60 (2)
					EBV+HHV-7	18-29 (1)
						41-60 (1)
**MS**	Total	27	F (23) / M (4)	52.0 (47.0-56.0)		
	DNA negative	26	F (22) / M (4)	51.5 (46.8-56.0)		
	DNA positive	1	F	53.0 (53.0-53.0)		
					HSV-2	41-60 (1)

Supplementary Table 3: Prevalence of HHV positivity by time in people with ME/CFS and healthy controls. Borderline significant results (0.05<p<0.1) are shown in **bold.**

		Prevalence (%)			Chi-square	
		HC	ME/CFS_ MM	ME/CFS_ SA	*P value* HC vs MM	HC vs SA
**HSV-1**	M1	6.25	7.14	6.25	0.9221	>0.999
	M2	6.67	0.00	12.50	0.3255	0.5830
	M3	0.00	0.00	0.00	n/a	n/a
	M4	20.00	10.00	6.25	0.5040	0.2538
	M5	0.00	0.00	0.00	n/a	n/a
	M6	7.14	0.00	13.33	0.4123	0.5844
**EBV**	M1	56.25	28.57	37.50	0.1269	0.2879
	M2	40.00	42.86	25.00	0.8759	0.3719
	M3	42.86	38.46	25.00	0.8163	0.3006
	M4	40.00	50.00	43.75	0.6217	0.8325
	M5	40.00	50.00	26.67	0.6217	0.4386
	M6	28.57	33.33	33.33	0.8086	0.7818
**HHV-6B**	M1	25.00	28.57	56.25	0.8253	**0.0719**
	M2	33.33	28.57	37.50	0.7818	0.8085
	M3	14.29	46.15	43.75	**0.0700**	**0.0789**
	M4	26.67	50.00	50.00	0.2338	0.1826
	M5	13.33	40.00	40.00	0.1262	**0.0986**
	M6	28.57	22.22	26.67	0.7350	0.9087
**HHV-7**	M1	93.75	92.86	87.50	0.9221	0.5442
	M2	88.67	100.00	87.50	0.1568	0.9449
	M3	85.71	92.31	87.50	0.5860	0.8859
	M4	93.33	100.00	87.50	0.4047	0.5830
	M5	93.33	90.00	86.67	0.7634	0.5428
	M6	85.71	100.00	86.67	0.2354	0.9408

Supplementary Table 4. HHV viral load in saliva of participants who were HHV DNA positive in the ddPCR screening assay of plasma and PBMC

	Participant ID (age/gender)	Detected HHV (sample type)	Median viral load (IQR, copies/ml saliva) for 6 months			
			HSV-1	EBV	HHV-6B	HHV-7
**HC**	6070 (18/F)	HHV-6B (plasma)	0	4080 (0-60300)	161700 (1208500-2889315)	29348 (18095-39050)
	8036 (44/F)	EBV (PBMC)	0	0 (0-58560)	0	10500 (8313-12556)
	13006 (42/F)	HHV-6B (plasma)	0	0 (0-8208)	0 (0-108)	0
	13018 (23/F) *	EBV (PBMC)	0	4500	0	26333
	13025 (32/F)	HHV-7/CMV (PBMC)	0	0	580 (0-790.4)	3900 (2691-4855)
**ME/CFS_MM**	1030 (42/F)	HHV-7 (PBMC)	0	0 (0-2850)	0 (0-660)	261300 (129183-576350)
	7079 (53/F)	EBV (PBMC)	0	80242 (10291-197375)	0	1917 (1423-3311)
**ME/CFS_SA**	6041 (35/F)	HHV-7 (PBMC)	0 (0-1068750)	0 (0-1319)	2097 (1698-2887)	37470 (23010-50297)
	6046 (32/F)	HHV-7 (PBMC)	0	14329 (0-424833)	0 (0-575.5)	22500 (15583-30427)
	6049 (55/F)	HHV-7 (PBMC)	0 (0-19755)	14738 (0-44250)	72 (0-489)	46287 (37275-61250)
	6052 (57/F)	HHV-7/EBV (PBMC)	0	0 (0-445)	0 (0-690)	13400 (9650-17550)
	6068 (18/F)	HHV-7/EBV (PBMC)	0	10800 (0-26152)	2023 (1652-2847)	50630 (36745-62950)

*One time visited

Supplementary Table 5. Age related HHV viral load in saliva

		n	EBV	P value	HHV-6B	P value	HHV-7	P value
			Median viral load (IQR, copies/ml saliva) for 6 months					
**HC**	UNDER 40	6	2040 (0.0-26225)	0.696	290.0 (0-405100)	0.036	27841 (4100 -37763)	0.147
	OVER 40	10	0.0 (0.0-7077)		0.0 (0.0 - 0.0)		7330 (661.3-19475)	
**ME/CFS_ MM**	UNDER 40	4	0.0 (0.0 - 0.0)	0.296	591.6 (145.8-770.5)	0.031	11571 (2633-35777)	0.142
	OVER 40	10	0.0 (0.0 - 146063)		0.0 (0.0 -0.0)		70375 (4192-103187)	
**ME/CFS_ SA**	UNDER 40	7	0.0 (0.0 - 10800)	0.677	2023.0 (0.0-2225)	0.132	23276 (13480 -50630)	0.516
	OVER 40	9	0.0 (0.0 - 7369)		0.0 (0.0 – 156.0)		13400 (2934-95087)	

Supplementary Table 6. Concurrent salivary HHV distribution over time

	HC (n=)	Month1	Month2	Month3	Month4	Month5	Month6
1	not detected	-	1	1	1	1	1
2	EBV only	1	1	1	-	-	-
3	HHV-6B ONLY	-	-	-	-	-	1
4	HHV-7 only	6	5	6	7	7	4
5	HHV-7+HHV-6B	-	2	1	1	1	3
6	HHV-7+EBV	5	2	4	1	5	4
7	HHV-7+HHV-6B+EBV	3	3	1	2	1	-
8	HHV-7+HHV-6B+HSV-1	1	-	-	-	-	-
9	HHV-7+EBV+HSV-1	-	-	-	2	-	-
10	HHV-7+HSV-1	-	1	-	-	-	1
11	HSV-1+ EBV	-	-	-	-	-	-
12	HSV-1+EBV+HHV-6B+HHV-7	-	-	-	1	-	-
		16	15	14	15	15	14

	ME/CFS_MM (n=)	Month1	Month2	Month3	Month4	Month5	Month6
1	not detected	1	-	-	-	1	-
2	EBV only	-	-	-	-	-	-
3	HHV-6B only	-	-	1	-	-	-
4	HHV-7 only	6	5	3	1	1	4
5	HHV-7+HHV-6B	3	3	4	2	3	2
6	HHV-7+EBV	2	5	4	4	4	3
7	HHV-7+HHV-6B+EBV	1	1	1	2	1	-
8	HHV-7+HHV-6B+HSV-1	-	-	-	1	-	-
9	HHV-7+EBV+HSV-1	1	-	-	-	-	-
10	HHV-7+HSV-1	-	-	-	-	-	-
11	HSV-1+EBV	-	-	-	-	-	-
12	HSV-1+EBV+HHV-6B+HHV-7	-	-	-	-	-	-
		14	14	13	10	10	9

	ME/CFS_SA (n=)	Month1	Month2	Month3	Month4	Month5	Month6
1	not detected	1	1	1	1	1	1
2	EBV only	1	-	1	-	1	-
3	HHV-6B only	-	-	-	-	-	-
4	HHV-7 only	4	6	6	5	4	7
5	HHV-7+HHV-6B	5	4	5	3	6	2
6	HHV-7+EBV	1	1	1	1	3	2
7	HHV-7+HHV-6B+EBV	3	2	2	5	-	1
8	HHV-7+HHV-6B+HSV-1	1	-	-	-	-	-
9	HHV-7+EBV+HSV-1	-	-	-	-	-	-
10	HHV-7+HSV-1	-	1	-	-	-	-
11	HSV-1+EBV	-	1	-	1	-	1
12	HSV-1+EBV+HHV-6B+HHV-7	-	-	-	-	-	1
		16	16	16	16	15	15

Supplementary Table 7. The R value of Spearman's analysis between HHV virus and ME/CFS symptom scores from ME/CFS_SA

Spearman r		HSV-1	EBV	HHV-6B	HHV-7	PEM	Sleepdysf	Pain	Neurocog	Autonomic	Neuroendo	Immune
**ME/CFS_SA-pattern 1 Fluctuating repertoire (n = 8)**	HSV-1	1										
	EBV	-0.033	1									
	HHV-6B	0.055	0.096	1								
	HHV-7	0.102	0.253	0.167	1							
	PEM	0.232	-0.022	0.284	0.202	1						
	Sleepdysf	-0.122	-0.182	0.472***	-0.202	0.120	1					
	Pain	0.158	0.100	0.522***	0.491***	0.646***	-0.010	1				
	Neurocogn	0.117	0.046	0.650***	0.443**	0.414**	0.308*	0.746***	1			
	Autonomic	0.168	0.205	0.496***	0.472***	0.524***	0.038	0.835***	0.666***	1		
	Neuroend	-0.034	-0.180	0.460**	0.053	0.105	0.724***	0.147	0.263	0.294*	1	
	Immune	0.243	-0.021	0.407**	0.060	0.480***	0.156	0.719***	0.410**	0.762***	0.335*	1
**ME/CFS_SA-pattern 2 Stable repertoire (n = 7)**	HSV-1	1										
	EBV	0.597***	1									
	HHV-6B	-0.200	-0.261	1								
	HHV-7	-0.412**	-0.453**	0.176	1							
	PEM	-0.016	0.288	-0.240	-0.324*	1						
	Sleepdysf	0.146	0.256	-0.033	-0.103	0.251	1					
	Pain	0.160	0.476**	-0.594***	-0.055	0.467**	0.322*	1				
	Neurocogn	0.260	0.401**	-0.431**	-0.306*	0.358*	0.213	0.557***	1			
	Autonomic	0.316*	0.389*	-0.250	-0.170	0.246	0.697***	0.354*	0.429**	1		
	Neuroend	0.005	0.262	-0.415**	-0.146	0.549***	0.538***	0.624***	0.556***	0.611***	1	
	Immune	0.088	0.183	-0.157	-0.145	0.388*	0.169	0.471**	0.590***	0.298	0.388*	1
**ME/CFS_ acute illness episode (ME/CFS_SA ; n=6, ME/CFS_MM; n=1)**	HSV-1	1										
	EBV	-0.111	1									
	HHV-6B	0.292	0.139	1								
	HHV-7	-0.306	0.003	0.548**	1							
	PEM	-0.409	-0.428*	-0.647**	-0.089	1						
	Sleepdysf	0.205	0.504*	0.454*	0.211	-0.321	1					
	Pain	-0.016	-0.203	-0.426*	-0.226	0.625**	0.258	1				
	Neurocogn	0.325	0.112	0.059	-0.382	-0.294	0.314	0.239	1			
	Autonomic	-0.179	0.204	-0.195	0.019	0.300	0.381	0.380	0.130	1		
	Neuroend	-0.260	0.306	0.268	0.315	-0.066	0.526*	0.133	0.070	0.517*	1	
	Immune	0.228	-0.384	-0.122	-0.043	0.597**	0.140	0.564**	-0.063	0.376	0.129	1

Supplementary Figures

ME/CFS RESEARCH PROJECT
SYMPTOMS ASSESSMENT – DAY 1 of acute illness or significant worsening of symptoms

Sex: Male ☐ Female ☐	Project ID Nº (For LSHTM use only)
Date of birth: ____/____/________ (dd/mm/yyyy)	Today's date: ____/____/________ (dd/mm/yyyy)

A. Please describe your current illness or worsening of symptoms.

____

____

____

____

1. How much time during the past week...

	None of the time	A little of the time	Some of the time	A good bit of the time	Most of the time	All of the time
Did you feel worn out?	0	1	2	3	4	5
Did you have a lot of energy?	0	1	2	3	4	5
Did you feel tired?	0	1	2	3	4	5
Did you have enough energy to do the things you wanted to do?	0	1	2	3	4	5
Did you feel full of pep (lively)?	0	1	2	3	4	5

2. Please mark the line with an 'X' to describe the severity of pain that you are currently experiencing (think of the last week including today), from no pain at all (mark on the left of the line) to maximum pain (mark on the right of the line).

No pain ______________________________ **Pain as bad as possible**

3. Please mark the line with an 'X' to describe the severity of fatigue that you are currently experiencing (think of the last week including today), from no fatigue at all (mark on the left of the line) to maximum fatigue (mark on the right of the line).

No fatigue ______________________________ **Fatigue as bad as possible**

Please mark the next two lines thinking about how much of that fatigue is physical and how much is mental.

No physical fatigue ______________________________ **Physical fatigue as bad as possible**

No mental fatigue ______________________________ **Mental fatigue as bad as possible**

4. Please tick the boxes using the scale above them to tell us more about which of the symptoms listed below you have had over the past week:

	**Absent**	**Mild**	**Moderate**	**Severe**
1. Sore throat				
2. Symptoms of flu				
3. Fever or chills				
4.Tender glands in the neck or arm pit				
5. Viral infections				
6. Sensitivities to food, medications, chemicals, smells/odours, and/or other				
6.a. Intolerance to alcohol (alcohol makes you feel ill/much worse)				
7. Feeling ill (malaise) after exertion/activity which lasts for a long time (more than 24 hours)				
8. Pain after exertion/activity which lasts for a long time (more than 24 hours)				
9. Muscle pain				
10. Muscle discomfort				
10.a. Muscle twitching				
11. Stiffness in the mornings				
11a. Pain in chest or abdomen				
12. Pain in two or more joints, without swelling or redness				
13. Joint pains moving to different joints without swelling or redness				

14. Neck weakness				
15. Back weakness				
16. Air hunger, difficulty in breathing or shortness of breath on exertion/activity				
17. Muscle weakness				
18. Headaches which are new, different or worse than before the disease started				
19. Migraine which is different/worse than before				
20. Unusual sensitivity to light and/or noise				
21. Temporary disturbance in eyesight				
22. Tingling or numbness in arms and/or legs				
23. Loss of balance, unsteadiness on feet when standing, or inability to focus the vision				
24. Poor coordination or unsteady movements (unsteadiness on walking)				
25. Short term memory problems				
26. Trouble concentrating				
27. Confusion or 'brain fog'				
28. Disorientation				
29. Difficulty understanding things, thinking clearly				
29.a.Difficulty finding or saying words				
30. Difficulty retaining/ recalling information				
31. Slow thinking				
31a. Difficulty making decisions				
32. Unrefreshing sleep				
33. Problems in sleep quality or duration (other than sleep apnoea), such as insomnia, changing night for day, awakening during the night				
34. Marked physical or mental fatigue or exhaustion after **minimal** exertion/activity, which lasts for a long time (more than 24 hours)				
35. Fatigue or exhaustion after levels of activity that should normally not cause fatigue (i.e. would not cause fatigue before you got ill)				
36. Intolerance to exercise (it is difficult to exercise)				
37. Worsening of symptoms after exertion/activity, which lasts for a long time (more than 24 hours)				
38. Worsening of symptoms with stress				

Page 3

39. Intolerance to (difficulty with) standing on your feet				
40. Dizziness while standing up				
41. Palpitations while **standing up**				
41a. Palpitations (feeling that your heart is racing or pounding)				
42. Feeling light-headed				
43. Being extremely pale				
44. Being unusually sweaty				
44a. Unusually cold hands or feet				
45. Intolerance to extremes of heat and cold				
46. Feeling sick (nausea)				
46. a. Irritable bowel symptoms, such as diarrhoea or constipation, abdominal pain, and bloating				
47. Bladder problems, such as having a sudden need to urinate (pass water), urinating more often than usual, or waking up during the night to pass water				
48. Decreased sexual interest and/or function				
49. Abnormal appetite or significant changes in weight (not intentional)				

END OF FORM - **If you have any queries, please phone the research nurse on 020 7XXX XXXX and she will be happy to help you.**

Thank you for continuing to participate!

Page 4

Supplementary Figure 1. Clinical symptom assessment forms. The form was completed by the participant at the time that each saliva sample was collected and returned, i.e. at Month 1, 2, 3, 4, 5 and 6 and then there was an disease exacerbation episode.

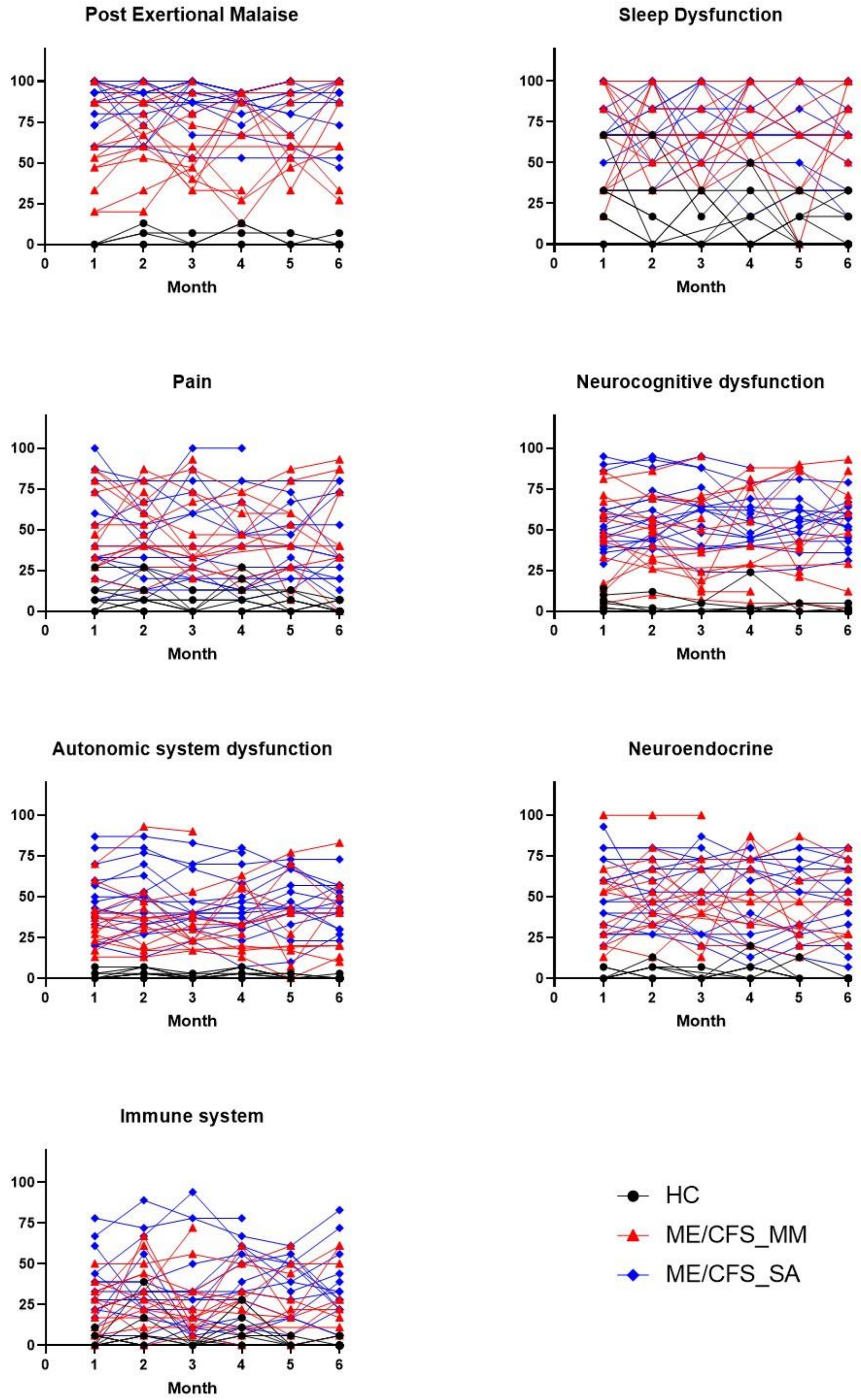

Supplementary Figure 2: Reported symptoms at monthly intervals in participants with mild/moderate (red) or severe (blue) ME/CFS or from healthy controls. Self-reported symptoms were submitted via questionnaire, with weighted responses determined under each symptom domain for each participant at each time point, on a scale of 1 – 100.

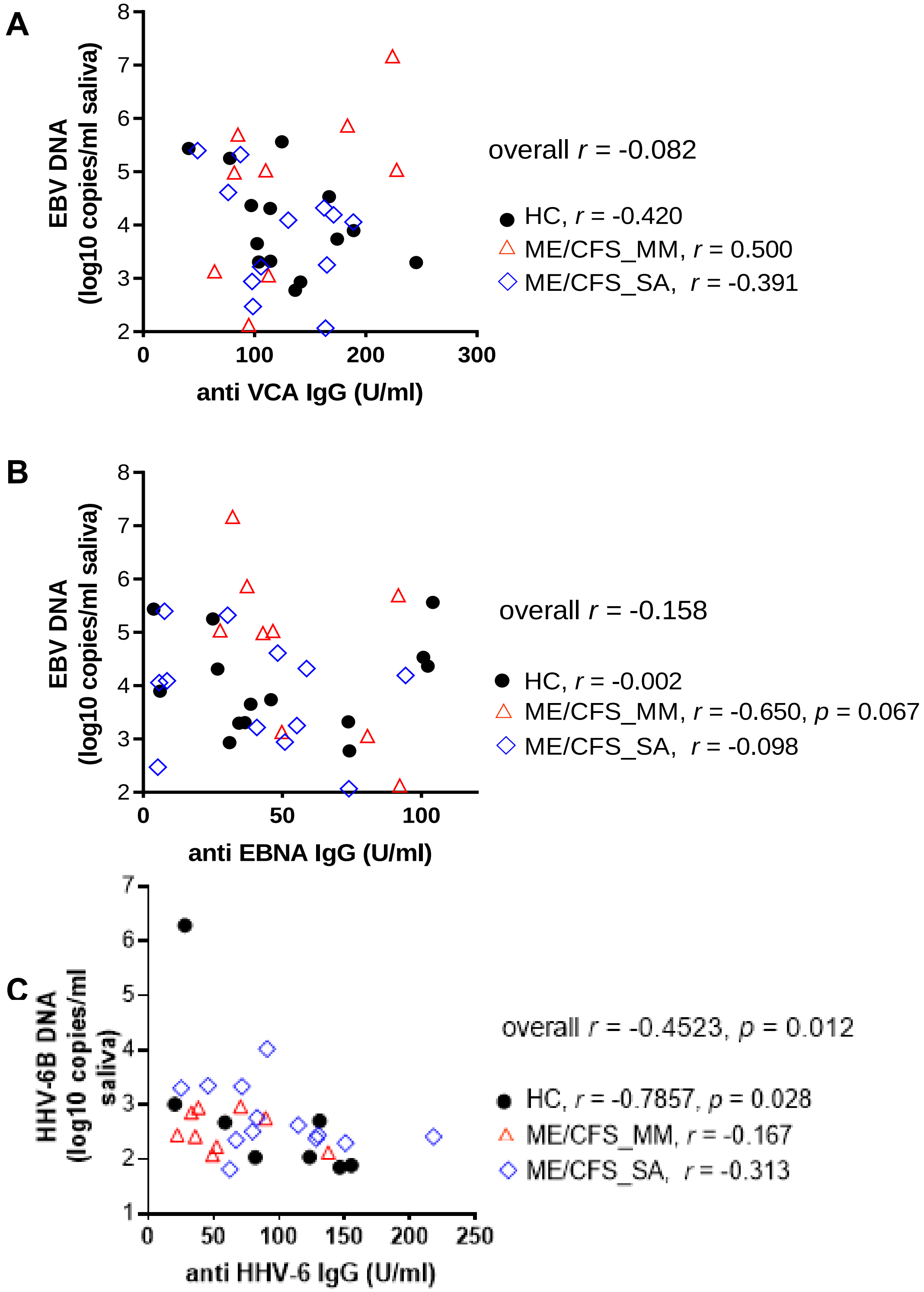

Supplementary Figure 3. Correlation analysis between salivary HHV viral DNA concentration and anti HHV IgG titre. (A) EBV viral DNA concentration with anti VCA IgG (B) EBV viral DNA concentration with anti EBNA IgG (C) HHV-6B viral load with anti HHV-6 IgG. Average viral DNA concentration measured monthly for 6 months was analysed with previously measured plasma anti HHV IgG level. Spearman's coefficient was denoted as *r*. *, $p < 0.05$. Zero DNA concentration values are not shown due to the logarithmic values but were included in the analysis.

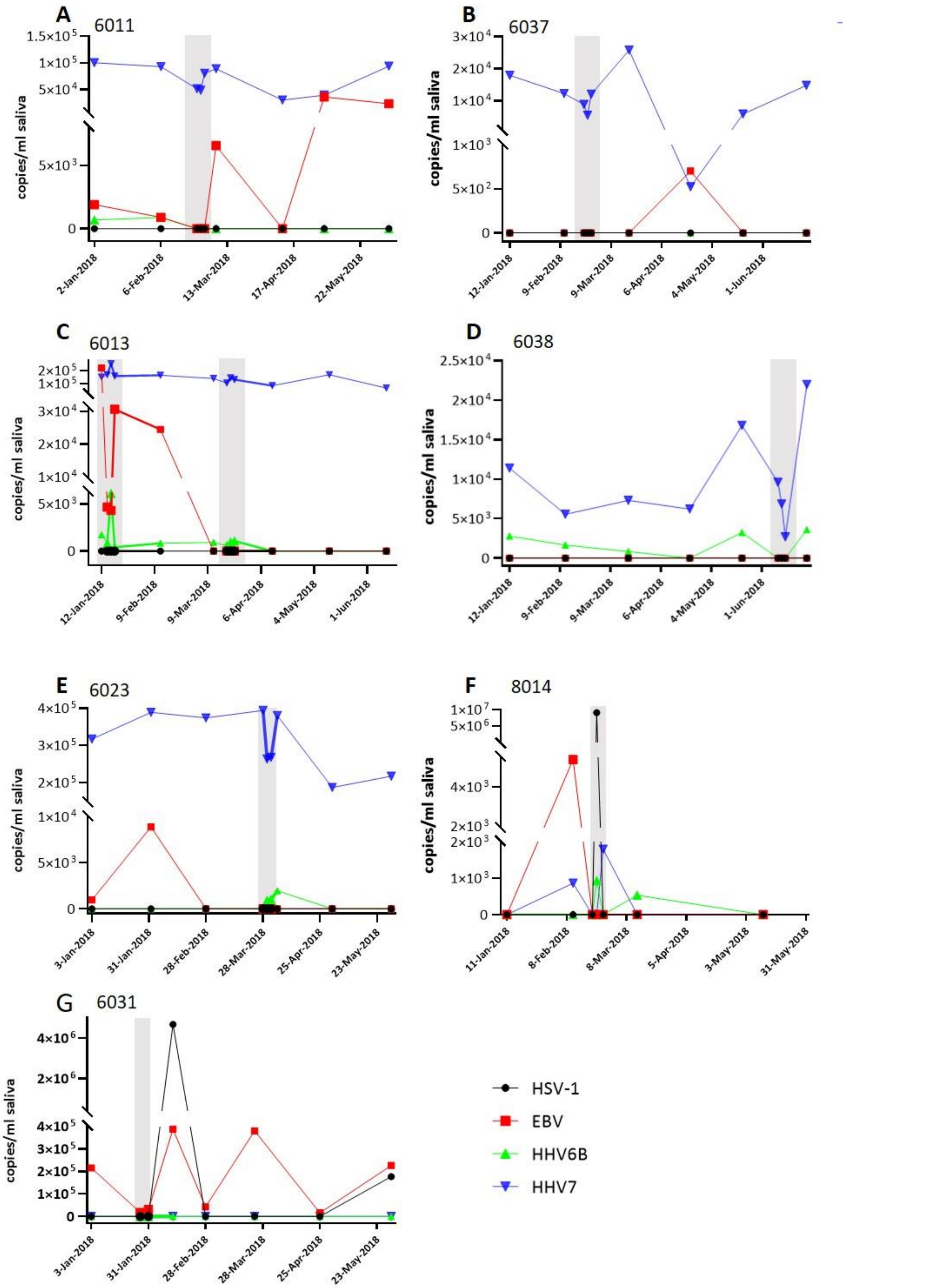

Supplementary Figure 4. Change of HHV viral DNA concentrations in people with ME/CFS (n=7) who had acute illness or worsening disease severity (highlighted in grey).

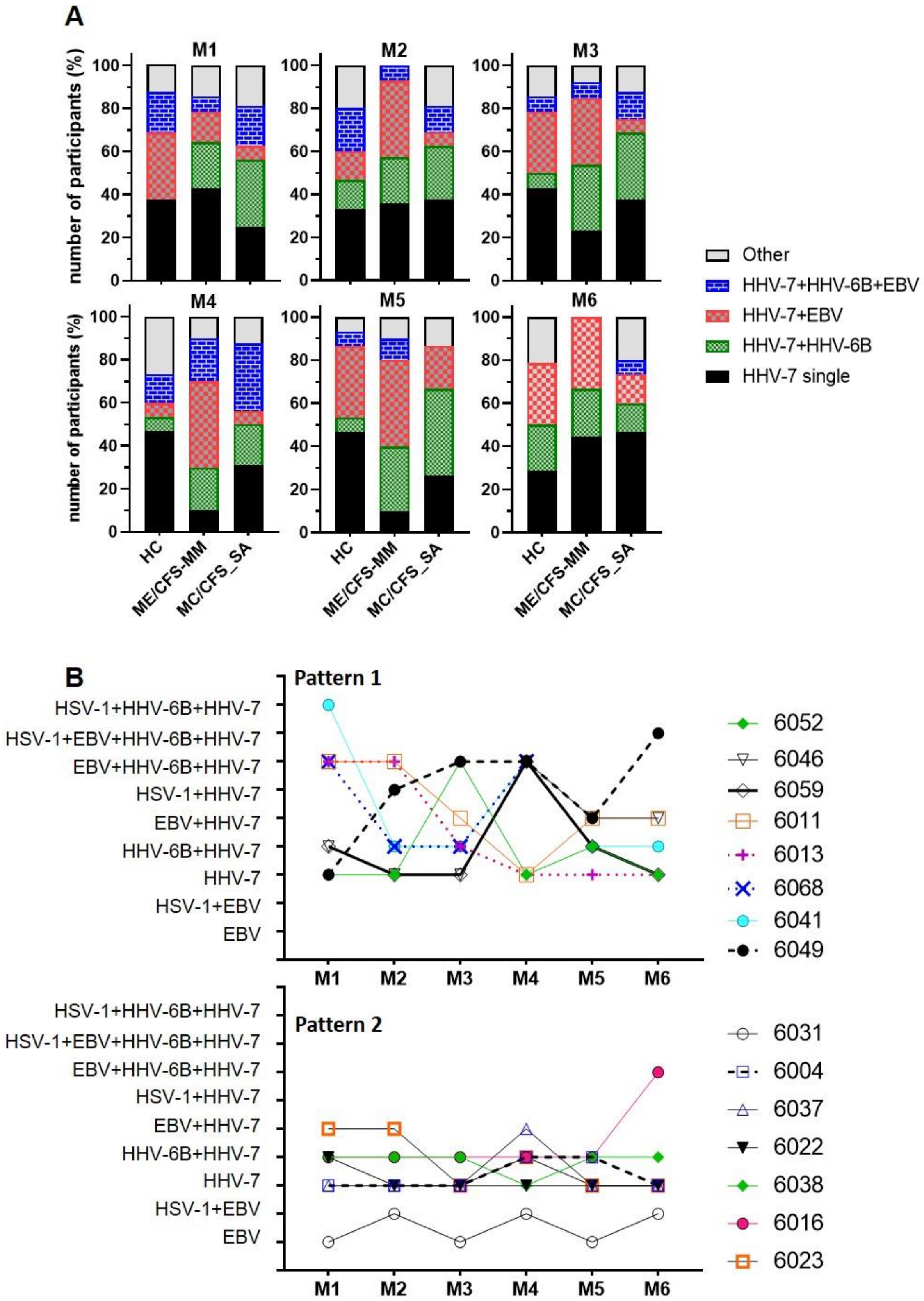

Supplementary Figure 5. Concurrent salivary HHV distribution over time. A) Distribution of different combinations of HHV DNA detected in saliva from participants with mild/moderate (MM) or severe (SA) ME/CFS and healthy controls (HC). B) Severely affected ME/CFS patients had either fluctuating HHV DNA repertoires (Pattern 1) or more constant repertoires (Pattern 2).

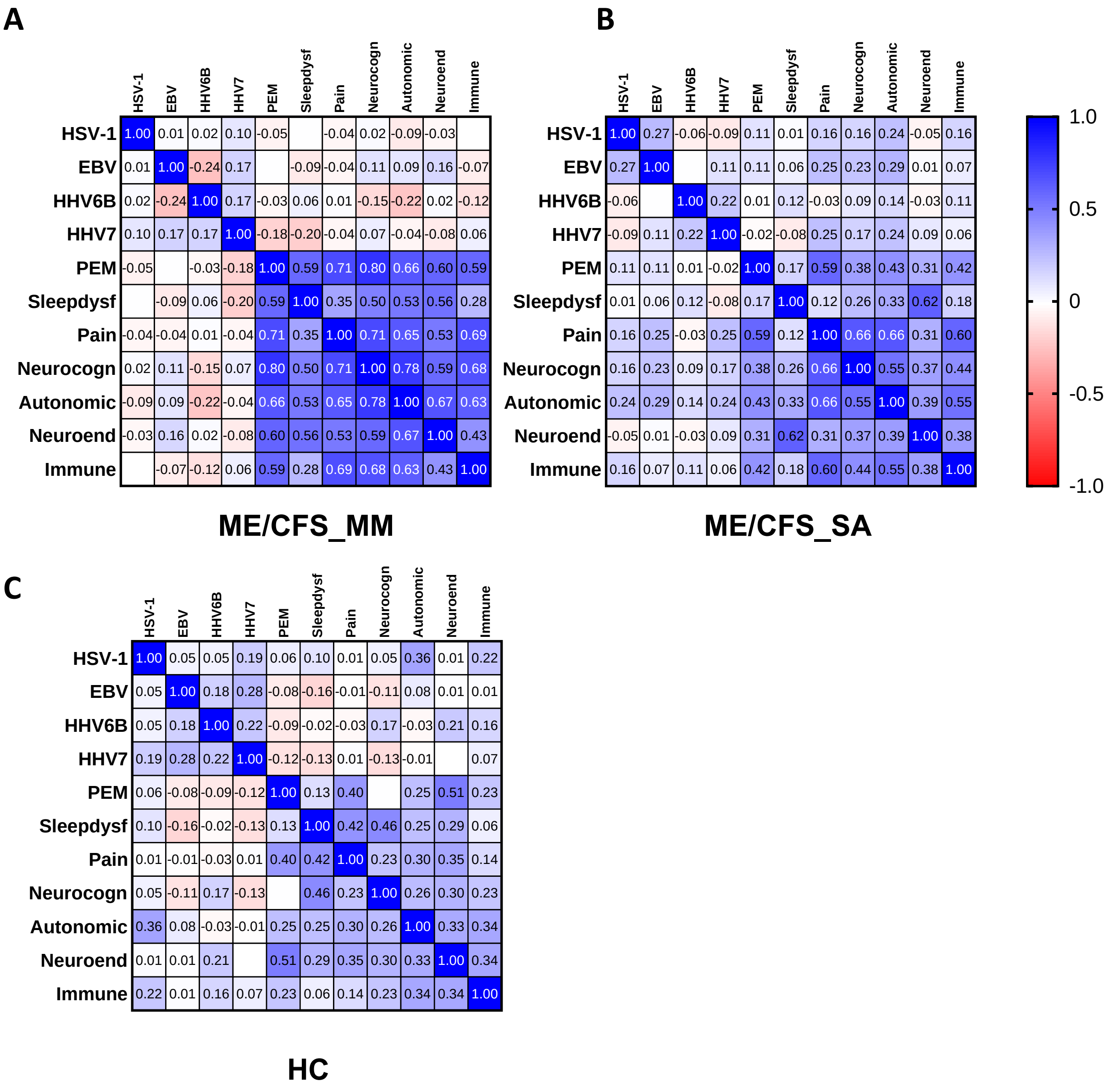

Supplementary Figure 6. Correlation matrixes of seven typical symptoms domains of ME/CFS with HHV viral DNA concentration in saliva from people with ME/CFS. Correlation efficient between HHV viral loads and symptoms scores across 6 months in (A) ME/CFS_MM, (B) ME/CFS_SA and HC (C)

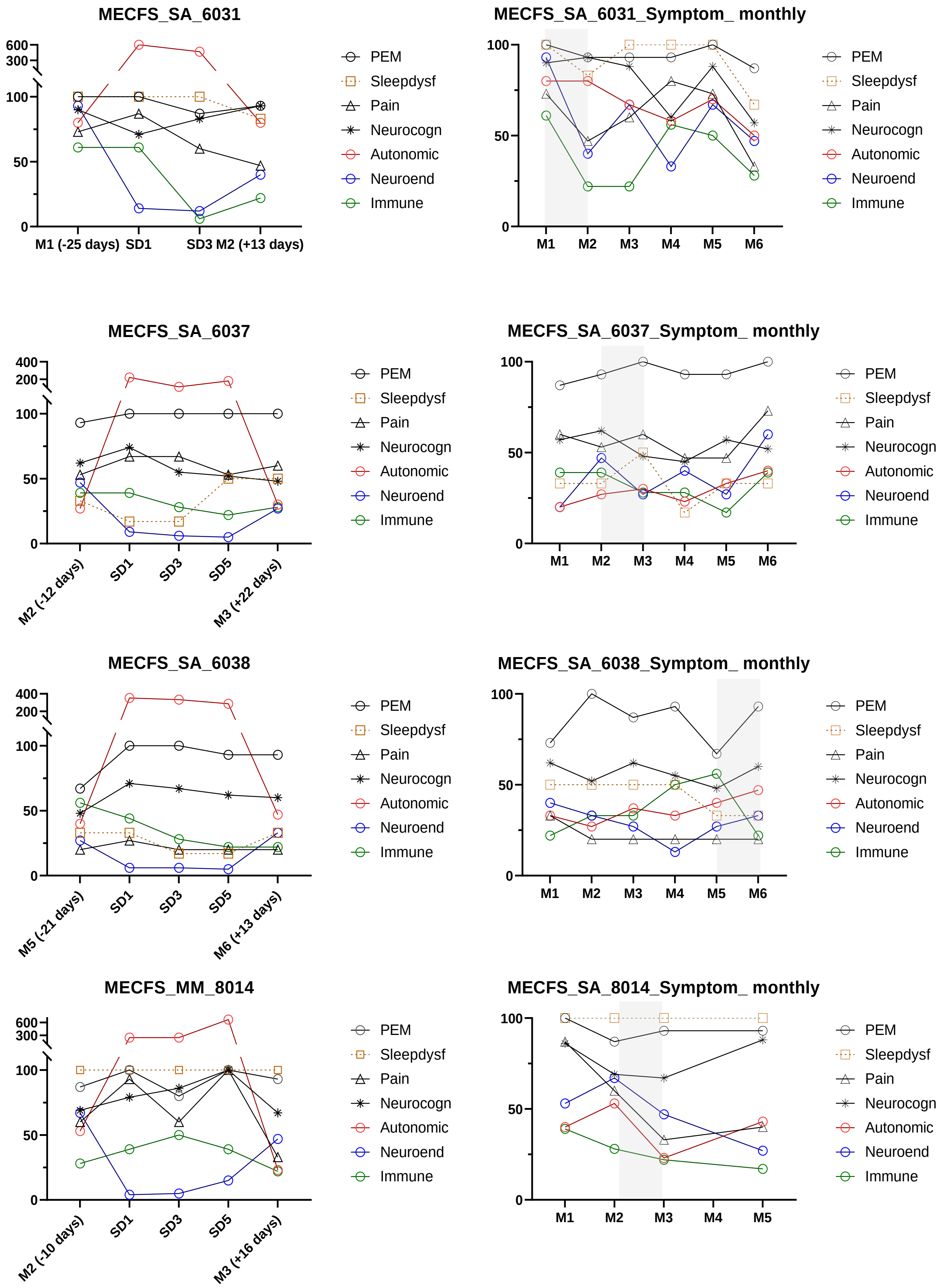

Supplementary Figure 7. Change of symptoms score in people with ME/CFS (n=7) who had acute illness or worsening of disease symptoms. Sick days (left panel) and monthly (right panel). Sick days occurred in the light grey area.
